# The Importance of Microorganisms for Sustainable Agriculture—A Review

**DOI:** 10.3390/metabo12111100

**Published:** 2022-11-11

**Authors:** Marcel Antoszewski, Agnieszka Mierek-Adamska, Grażyna B. Dąbrowska

**Affiliations:** Department of Genetics, Faculty of Biological and Veterinary Sciences, Nicolaus Copernicus University, Lwowska 1, 87-100 Toruń, Poland

**Keywords:** microbiome, PGPM, plant fitness, bioinoculants, symbiotic interactions, *Trichoderma*, stringent response, *RSH* genes, rhizosphere, alarmones

## Abstract

In the face of climate change, progressive degradation of the environment, including agricultural land negatively affecting plant growth and development, endangers plant productivity. Seeking efficient and sustainable agricultural techniques to replace agricultural chemicals is one of the most important challenges nowadays. The use of plant growth-promoting microorganisms is among the most promising approaches; however, molecular mechanisms underneath plant–microbe interactions are still poorly understood. In this review, we summarized the knowledge on plant–microbe interactions, highlighting the role of microbial and plant proteins and metabolites in the formation of symbiotic relationships. This review covers rhizosphere and phyllosphere microbiomes, the role of root exudates in plant–microorganism interactions, the functioning of the plant’s immune system during the plant–microorganism interactions. We also emphasized the possible role of the stringent response and the evolutionarily conserved mechanism during the established interaction between plants and microorganisms. As a case study, we discussed fungi belonging to the genus *Trichoderma*. Our review aims to summarize the existing knowledge about plant–microorganism interactions and to highlight molecular pathways that need further investigation.

## 1. Introduction

Traditional agricultural techniques, such as chemical fertilizers, pesticides, fungicides, and herbicides, enable the protection of crop plants against pathogens and ensure better yield. Chemical compounds present in agricultural chemicals are harmful to the environment and cause soil, atmosphere, and water pollution [1]. These compounds are the reason for the extinction of fish [2], bees [3], and plants [4,5], and pose a threat to the biodiversity of soil bacterial [6,7,8] and fungal communities [9]. Chemical plant protection products negatively affect agricultural soils, i.e., they change soil physical properties, (e.g., texture, permeability, porosity), they disturb the cycle of the elements, such as phosphorus and nitrogen, and they decrease the complexity of soil microbiome [10]. In the face of a growing world population and increased demand for food both in terms of food quantity and food quality, the usage of bioinoculants, i.e., biofertilizers to increase the yield and biopesticides to protect plants, is the future of agriculture. Bioinoculants comprised of living or dormant microbes that are able to promote plant growth and development are called PGPM (plant growth-promoting microorganisms) and have great potential not only for enhancing plant yield but also for remediation of degraded soils [11,12,13]. Bioinoculants are cost-effective and environmental-friendly approaches in agriculture [14]. The first step in a bioinoculant formulation is the isolation and identification of a microbe. The further potential of a particular microorganism for plant growth promotion needs to be verified, and this ability should be confirmed in laboratory and field conditions. Moreover, potential risks to other organisms, such as animals and natural soil microbiomes, should be also determined [15]. The best-known examples of PGPM are mycorrhizal fungi and bacteria belonging to *Rhizobium;* however, plant growth-promoting microorganisms are found among varied taxa of bacteria, fungi, and algae [16]. In Table 1, several examples of PGPM are shown.

One of the major drawbacks of the application of bioinoculants is the fact that the number of bioinoculants tested in laboratory/greenhouse conditions fail in the field trials. This is mostly because microbes introduced to the environment have to compete for a niche with native microorganisms in order to attain sufficient abundance. The ability of introduced microbes to survive and thrive is variable and significantly depends on environmental conditions including temperature, rainfall, and soil type, as well as on interactions with the host plant and other organisms. A good example of this dependence is the field trial that showed that the promotion of plant growth by subtropical strains of *Azotobacter chroococcum* and *Azospirillum brasilense* was observed only when used in the same type of climate and not in the alpine region with a temperate climate [80]. Moreover, it was demonstrated that the high biodiversity of the native soil microbiome has a negative impact on the survivability of applied bioinoculants, i.e., there is a negative correlation between the diversity of the soil microbes and the survival rate of the introduced strain [81]. Therefore, when the native microbiome biodiversity is low, the chance for a new strain to thrive is higher which corresponds with better availability of nutrients and reduced competition among microorganisms for niche [82,83,84]. Moreover, several studies showed that PGPM that promotes the growth of a particular plant species might not be beneficial for other species of plants. For example, fungi belonging to *Penicillium* sp. and *Trichoderma* sp. showed diversified effectiveness in enhancing the growth of varied wheat (*Triticum aestivum* L.) cultivars [85]. Similarly, the effect of inoculation of winter and spring varieties of oilseed rape (*Brassica napus* L.) with different strains of PGPM on the germination rate and growth of seedlings depended on the plant variety [86]. On the other hand, different strains of the same microbial species might differ significantly in their ability to promote plant growth and development. For example, Znajewska et al. [59] showed that seven *Trichoderma viride* isolates had various effects on winter rapeseed germination and growth promotion depending on the used strain. 

Microorganisms colonize not only roots but also other plant tissues and organs including stems, leaves, flowers, seeds, and fruits. The aerial part of plants colonized by microbes is called the phyllosphere, whereas the rhizosphere is the soil adjacent to the root [87]. In contrast to the rhizosphere, the above-ground parts of plants are scarce in water and nutrients. Only a small number of microorganisms that reach the surface of the plant will land on beneficial spots and will have conditions to survive [88]. As a consequence, the number of microorganisms living in the rhizosphere is much higher than in the phyllosphere. Microbes are present at every stage of plant development, from seed to fully developed plant producing a new generation of seeds [89]. Some microorganisms live on the surfaces of plant organs, i.e., epiphytes whereas others are able to colonize the internal tissues of plants, i.e., endophytes [90,91]. 

**Figure 1:**
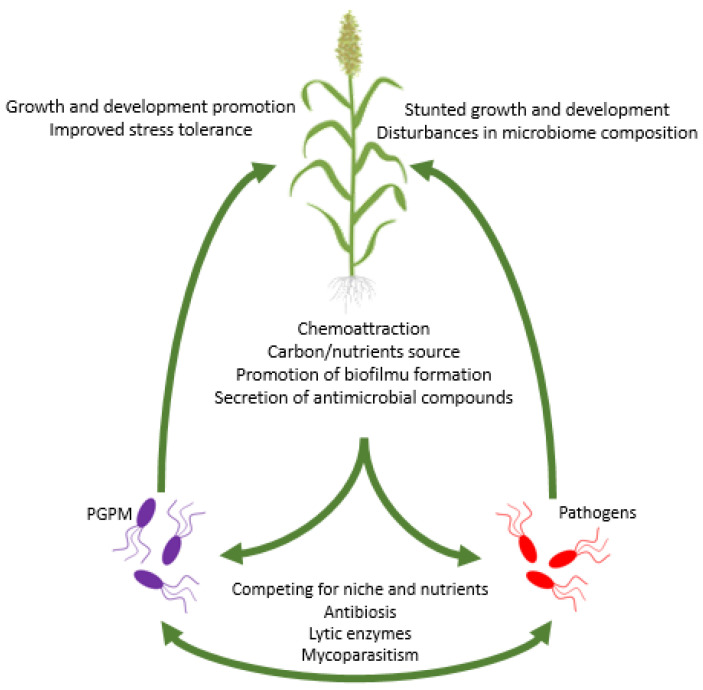
Multidimensional network of interactions between plants and PGPM, between plants and pathogens, and between PGPM and pathogens.

Plant growth-promoting microorganisms stimulate plant growth and development through various direct and indirect mechanisms (Figure 1, Table 1). Production of phytohormones [92,93], nitrogen assimilation [94], solubilization, and mineralization of macro- and micro-elements [95,96], and modulation of the endogenous level of ethylene in plants tissues [97] are examples of direct mechanisms. Examples of indirect mechanisms are inhibition of pathogens growth through antibiosis [98], secretion of lytic enzymes [99], and competition, e.g., via siderophores production [100], induction/inhibition of plant genes expression [101], induction of plant immune response [102], and manipulation of plant microbiome composition [103]. This work aims to summarize the current knowledge about the interactions of plants with beneficial microbes, and how those interactions affect the overall health of the plant. For further development of environmental-friendly methods of plant cultivation, is it crucial to deeply understand the molecular mechanisms underneath (i) the recruitment of useful microbes by plants, (ii) the interactions among microorganisms, and (iii) the plant-microorganism interplay. The interactions between plants and microorganisms can be divided into three types, i.e., interactions are either neutral, negative, or positive in their effects on the host plant. This review focuses exclusively on positive interactions and mechanisms underneath those interactions. In this work, we also discuss the role of stringent responses in interactions between plants and microorganisms.

## 2. Rhizosphere and Root Exudates

The rhizosphere is defined as “the field of action or influence of a root”, i.e., it is soil adjacent to the roots which are influenced by root exudates that are the mixture of several compounds produced and secreted by roots [104]. The main components of root exudates are water, enzymes, amino acids, nucleotides, vitamins, organic acids, fatty acids, sugars, phenolic compounds, anions, volatile organic compounds (VOCs), polysaccharides, and proteins [105,106,107,108,109,110,111,112]. The composition of exudates varies depending on the plant species, and even phenotype; it changes during plant development [107,113] and it is dependent on environmental conditions, such as temperature [114], light [115], the amount of nutrients [116,117,118,119], and stress factors [120,121,122]. Moreover, the type and composition of soil can also affect the composition of root exudates [123]. For instance, plants that grow in soil deprived of nitrogen probably do not secrete extra amino acids or proteins to the rhizosphere [119]. Root exudates are also an important source of elements present in the soil. It was estimated that about 10–44% of carbon compounds [124] and about 10–16% of nitrogen compounds [125] synthetized by the plant is secreted to the rhizosphere. In legumes, the rhizodeposition of nitrogen is estimated between 4% and 70%, depending on the plant species [126]. 

The rhizosphere is the hotspot of plant–microorganism interactions. Interactions between those organisms have a direct influence on the availability of soil nutrients for plants [95,127,128,129,130] and on plant tolerance toward biotic and abiotic stresses [131,132,133,134,135]. Exudates are a rich source of carbon and other nutrients and, therefore, the abundance of microorganisms in the rhizosphere can be up to a hundred times greater than in the bulk soil [136]. Moreover, root exudates allow plants to communicate with rhizosphere microorganisms and affect their behavior through the secretion of various signaling molecules [137,138,139,140]. For instance, barley (*Hordeum vulgare* L.) in response to infection by the fungus *Pythium ultimum,* secretes increased amounts of organic and phenolic acids, which activates the expression of the *phlA* gene in the endophytic bacterium *Pseudomonas fluorescens* CHA0. Hydroxymethylglutaryl-CoA synthase encoded by the *phlA* gene is involved in the synthesis of DAPG (1,4-diacetylphloroglucinol) that has antifungal activity [98]. Zhang et al. [141] demonstrated the role of organic acids in root exudates of cucumber (*Cucumis sativus* L.) and banana (*Musa acuminata* Colla). The citric acid present in cucumber root exudates attracted *Bacillus amyloliquefaciens* (isolated from cucumber rhizosphere) and *B. subtilis* (isolated from banana rhizosphere). Moreover, it induced the formation of the *B. amyloliquefaciens* biofilm. Fumaric acid present in banana root exudates promoted biofilm formation of both tested strains. Biofilm is a coherent multicellular structure embedded in a self-produced extracellular matrix that can be formed on the surface of plant organs. Members of biofilm are better protected from environmental factors; their nutritional status is enhanced and, therefore, the survival rate of members of biofilm is much higher than that of single bacteria cells, as reviewed in [142]. Another example of a molecule allowing plant–PGPM communication is polyamines present in root exudates, which inform rhizospheric microorganisms about the presence of a potential host plant [143]. For instance, putrescine and its precursor arginine attract *Pseudomonas* sp. and trigger a lifestyle change, promoting attachment to the root and formation of biofilm [144]. However, the best-known example is the mechanism of communication between *Rhizobium* and legumes. Flavonoids secreted by plants activate bacterial *nod* genes lead to secretion by bacteria of the nod factor. The nod factor promotes the formation of nitrogen-fixing nodules in roots [145,146].

Root exudates serve as a chemoattractant, by which plants “recruit” microorganisms (Figure 1). Several studies showed that the composition of root exudates has an enormous effect on the composition of plant microbiome [147,148,149,150,151,152]. The composition of root exudates is specific for each plant species, which enables plants to attract a particular set of microbes [153]. Moreover, plants can secrete substrates that are available only for selected microbial groups or compounds that are toxic for certain groups of microorganisms in order to inhibit their growth [154]. A prominent example is the amino acid canavanine, which is present in seeds and root exudates of legumes. Canavanine is toxic to a number of soil bacteria excluding rhizobia which possess the *msiA* gene encoding canavanine exporter that warrants canavanine resistance [155]. Mardani-Korrani et al. [156] demonstrated that canavanine is secreted by *Vicia villosa* Roth, significantly decreased the diversity and changed the composition of microbial communities in soil. The presence of canavanine caused an increase in the abundance of Firmicutes and Actinobacteria and decreased the number of Proteobacteria and Acidobacteria. Another example of the selective action of compounds present in root exudates is coumarin secreted by *Arabidopsis thaliana* (L.) Heynh. Coumarin selectively inhibits the proliferation of some pathogenic fungi, stimulates the growth of some *Pseudomonas* spp. and other microbes that belong to the PGPM group, and increases the bioavailability of iron by ferric ions reduction [157]. Coumarins are used by plants to increase the bioavailability of iron. Coumarins, such as scopoletin and esculetin, enable the mobilization of Fe from minerals in acidic and alkalic soil [158]. Moreover, the depletion of Fe in soils enhances the biosynthesis and secretion of coumarins in *A. thaliana* [157]. It is also worth noting that fungi also produce and secrete exudates that might affect other rhizosphere microorganisms. An interesting observation was made by Toljander et al. [159], who showed that arbuscular mycorrhizal fungi (AMF) affect the composition of bacterial community through mycelia exudates. Analysis of exudates produced by *Glomus* sp. showed that the main components are water, low molecular weight sugars, and organic acids. Moreover, it was shown that *Glomus* exudates inhibited the growth of several bacterial strains including opportunistic pathogens *Flavobacterium* spp. and increased the abundance of bacteria belonging to Gammaproteobacteria.

Root exudates are usually secreted without energy, mostly through diffusion, ionic channel, and vesicle transport. Among several transporters involved in the secretion of exudates by roots are ABC (ATP-binding cassette) transporters which transport lipids and flavonoids [160], and anion channels are involved in secreting carbohydrates [161]. Moreover, for the secretion of root exudates transmembrane proteins aquaporins (AQPs) that are related to the membrane reflection coefficient and root hydraulic conductivity seem to be of great importance, as reviewed in [162]. Aquaporins are present in endogenous and exogenous membranes of eukaryotes and prokaryotes and are responsible for symplastic transport of not only water but also low molecular weight compounds and non-charged molecules including urea, glycerol, hydrogen peroxide, ammonium ions, and some elements, e.g., silicon and boron, as reviewed in [163]. Interestingly, it was demonstrated that during ectomycorrhiza formation the expression of fungi *Laccaria bicolor* aquaporins significantly increased. Moreover, fungal aquaporins exhibit high permeability for NH_3_ and, therefore, it was suggested that they are involved in the transfer of this compound from fungal cytoplasm to plant [164]. *Glycine max* L. noduline 26 aquaglyceroporin (GmNod26) is located in the symbiosome membrane in N_2_-fixing nodules and is a transporter of ammonia. The C-terminal domain of GmNod26 interacts with the main ammonia assimilatory enzyme, i.e., glutamine synthetase, and this probably supports the effective assimilation of fixed nitrogen [165,166]. The potential of root exudates to determine the composition of the rhizosphere microbiome is also important for the biodegradation of various soil pollutants. Environmental pollution is a serious global challenge and needs urgent development of effective methods of remediation. An interesting observation was presented by Janczak et al. [167,168] who showed that the presence of plants had a positive effect on bacterial, (i.e., *Arthrobacter sulfonivorans,* and *Serratia plymuthica*), and fungal, (i.e., *Clitocybe* sp. and *Laccaria laccata*), ability to degrade polymers including polylactide (PLA) and poly(ethylene terephthalate) (PET). The effectiveness of biodegradation also significantly depended on the species, i.e., the presence of *Salix viminalis* L. (willow) enhanced the level of biodegradation more significantly that the presence of *B. napus* and *Miscanthus* x *giganteus* J.M.Greef, Deuter ex Hodk., Renvoize (giant miscanthus). It was suggested that root exudates probably support the growth of microorganisms and/or root exudates can activate microbial genes involved in the biodegradation of plastics, such as intra- and extra-cellular depolymerases. The breakdown of long polymers into oligo-, di-, and mono-mers enables uptake of those molecules by a bacterial cell which can be then utilized as a carbon and/or energy source [169]. Afzal et al. [170] reported that inoculation of Italian ryegrass (*Lolium multiflorum* Lam.) and birdsfoot trefoil (*Lotus corniculatus* L.) with alkane-degrading bacteria *Pantoea* ssp. and *Pseudomonas* sp. separately or in consortium resulted in higher biomass production by plants and bacterial consortium showed higher degradation ratio in comparison to single strain inoculants. The ability of these bacteria to degrade alkane was linked to the presence of genes encoding cytochrome p450 alkane hydroxylase (CYP153) and alkane monooxygenase (*alkB*). *Enterobacter ludwigii* possessing the CYP153 gene was able to degrade diesel fuel [171]. On the other hand, it was shown that the level of degradation of polycyclic aromatic hydrocarbon was reduced in the presence of ryegrass (*Lolium perenne* L.) root exudates [172] which suggest that plants might differ in their potential to enhance bioremediation potential of soil microorganisms. The PGPM are able to degrade various other compounds of different origins as a means for the promotion of plant growth and development. Allelochemical compounds secreted by plants can hinder the cultivation of other plant species in cropping systems. *Trichoderma harzianum* SQR-T037 was shown to degrade allelochemicals secreted by cucumber roots (4-hydroxybenzoic acid, vanillic acid, ferulic acid, benzoic acid, 3-phenylpropionic acid, and cinnamic acid). The use of strains able to biodegrade allelochemicals can ameliorate allelopathic stress in continuous cropping systems [173]. Among common soil contaminants, plant protection agents are of special interest since their persistence in the agricultural soil is high, and their concentration may increase with each application. Diuron, a phenylurea herbicide, has a mean half-life of 330 days. Inoculation of soil containing diuron with fungal endophyte *Neurospora intermedia* leads to degradation of 99% of diuron in the soil after 3 days. Moreover, the authors reported that this strain is able to degrade other phenylurea herbicides, e.g., fenuron, monuron, isoproturon, chlorbromuron, and chlortoluron [174]. Although organophosphorus pesticides are perceived as non-persistent, they are highly toxic for a wide variety of non-target organisms, including mammals and rhizospheric microbes. Tested strains of *T. harzianum* and *Metarhizium anisopliae* showed an ability to degrade a number of organophosphorus pesticides such as diazinon, profenofos, and malathion in a temperature range of 20–45 °C [175].

## 3. Microbiome and Holobiont

Plants provide a multitude of ecological niches for various organisms to thrive. All these organisms including bacteria, fungi, protists, and nematodes that live on the surface and inside tissues/organs of a certain plant form the plant microbiome [176]. Although each member of the microbiome might contain genes related to the promotion of plant growth and development, the expression of those genes is dependent on the composition of the whole microbiome, on the population dynamics of potential pathogens, and on environmental conditions [177]. For example, the consortium of six bacteria (*Arthrobacter nitroguajacolicus*, *Bacillus cereus*, *Bacillus megaterium*, *Bacillus mojavensis*, *Pseudomonas azotoformans*, and *Pseudomonas frederiksbergensis*) was much more effective in the protection of *Nicotiana attenuata* Torr. ex S.Watson (coyote tobacco) against fungal pathogens *Fusarium* sp. and *Alternaria* sp. than individual members of the consortium [178]. Analysis of *P. fluorescens* transcriptome in response to the presence of bacteria belonging to three different genera revealed significant differences in the transcription response of *P. fluorescens* to different competitors [179]. Moreover, another layer of microbiome complexity is added by the presence of microbial symbionts of the members of the plant microbiome. For example, plant-associated fungi are in symbiosis with bacteria, i.e., endofungal/endohyphal bacteria [180]. Interestingly, it was shown that endohyphal bacteria *Luteibacter* sp. significantly increases the production of indole-3-acetic acid (IAA) in fungi *Pestalotiopsis* sp. However, *Luteibacter* sp. is not able to synthesize IAA [181]. Viruses can also interact with the plant microbiome. *Dichanthelium lanuginosum* (Elliott) Gould (panic grass) grows in geothermal areas in consortium with its endophytic fungus *Curvularia protuberata*. When the fungus is infected with Curvularia thermal tolerance virus (CThTV), soil temperature tolerance of panic grass increases from 40 °C to 65 °C [182,183]. Those results clearly show that other microorganisms present in soil strongly affect the PGPM potential to promote plant growth and development.

Although plants recruit a number of diverse microorganisms, they show preferences toward specific bacteria species. Analysis of seeds of medicinal plant red sage (*Salvia miltiorrhiza* Bunge) collected at different locations revealed that there are no significant differences in the composition of the microbiome, whereas different plant species collected at the same location had significantly different microbiomes. This indicates that different species of plants exhibit a preference for specific groups of microorganisms [184]. Redford et al. [185] demonstrated the structure of microbial communities present on *Pinus ponderosa* Dougl. Ex C. Lawson needles are very similar regardless of geographic location. However, there are also examples of substantial differences in the microbiome of different cultivars of one plant species described in the literature. For example, Germida et al. [186] showed that root microbiomes of modern and older wheat cultivars were significantly different. Moreover, some microorganisms interact only with single plant species. For instance, a number of ectomycorrhizal fungi form mycorrhiza only with one species of a tree, e.g., *Suillus grevillei* and larch (*Larix* sp.) (as reviewed in [187]). Species of *Pinaceae* are the only hosts for the fungal genus *Rhizopogon* [188]. The most known example is the highly specialized relationship between legumes and rhizobia [189]. Wicaksono et al. [190], by studying bog ecosystems, found that microbiomes of vascular plants are less diversified than those of non-vascular plants including bryophytes. Specificity between host and microbes seems to be a plastic trait modulated by the environment [191]. The mechanisms underneath the preferences of the host plant toward a specific set of microorganisms are not well understood, but recently Salas-González et al. [192] showed that mechanisms involved in the maintenance of plant mineral nutrient homeostasis also contribute to microbiome assembly. 

The composition of the plant microbiome is largely dependent on the phase of plant growth and development. The mature seed is colonized by microorganisms that were associated with the mother plant, (i.e., microbes were transferred vertically), and those microorganisms are the first members of the microbiome of a newly emerging plant [193]. During germination, microbes transferred vertically have an advantage in plant colonization over the microorganisms present in the soil. During plant development, new symbiotic microorganisms transferred horizontally, (i.e., from the soil), appear [136,194]. Interestingly, it was demonstrated that microbes transferred vertically usually inhabit the phyllosphere, while the rhizosphere and root are colonized by the microorganisms from the soil. A study on oak (*Quercus robur* L.) microbial inheritance showed that microbial composition of the phyllosphere was very similar to the composition of the embryo [195]. Sánchez-López et al. [196] showed that seed endophyte *Methylobacterium* sp. Cp3 was transferred via seeds across three generations of the plant *Crotalaria pumila* Ortega. Moreover, when *Methylobacterium* sp. Cp3 was inoculated to the soil at the time of *C. pumila*, flowering migration of bacteria from soil to seeds was observed. Moreover, the microbiome strongly differs among plant organs and tissues, i.e., the surface of the leaf is colonized by different microorganisms in comparison to the rhizosphere microbes [177]. A study on native and cultivated *Agave* species showed differences in bacterial taxa colonizing the rhizosphere, the phyllosphere, and the leaf and root endosphere. Interestingly, a composition of the fungal microbiome was affected mainly by the host plant biogeography [197]. Zarraonaindia et al. [198] demonstrated that below- and above-ground microbial communities of grapevine (*Vitis vinifera* L.) were significantly different. Moreover, the composition of microbiomes of leaves, grapes, and flowers was more similar to the composition of soil microbiomes than to each other. Similarly, in sugarcane (*Saccharum* sp.), clear differences in microbial taxa between organ types were observed. Interestingly, the microbiome composition of the young shoots formed from the underground ratoon was very similar to the microbiome of roots [199]. The phyllosphere is a hostile and dynamic environment. Microbes present on plant above-ground surfaces are subjected to irregular nutrient availability and changeable environmental conditions. The phyllosphere microbiome seems to be even more dependent on environmental conditions than the root microbiome [88,185]. It should be pointed out that the data regarding phyllosphere microbiomes other than leaf microbiomes is still rather limited. A study on the microbiome of apple (*Malus domestica* Borkh.) flowers showed that the most popular are bacteria belonging to the extremophilic phylum *Deinococcus*-*Thermus*. Moreover, the composition of the flower microbiome was dependent on the phase of apple flower development [200]. 

The composition of the plant microbiome is also strongly influenced by various environmental factors, such as climate, soil properties [201,202,203], water [204], and nutrient availability [205,206]. The adaptation of plant metabolism to environmental change is strongly supported by rapidly changing microbial communities [204]. Analysis of the lettuce (*Lactuca sativa* L.) microbiome showed that the composition of the phyllosphere microbiome is strongly dependent on the time of the year [207]. Drought is one of the most important stress factors significantly affecting not only plant growth and the yield of crops [208], but also the plant microbiome. Drought affects the microbiome directly because a low level of soil moisture inhibits the growth of several microorganisms. In drought conditions, plants recruit stress microbiomes, i.e., the most beneficial group of microbes allowing the plant to adapt to a particular set of environmental conditions. For instance, the abundance of Actinobacteria increased in drought-treated roots and the rhizosphere of 18 species belonging to *Poaceae*. The results suggest that although the microbiome is species-specific, drought caused a relatively conserved response in different hosts [209]. In drought stress conditions, an increase in the abundance of the rhizospheric drought stress-resistant bacteria in *Oryza sativa* L. was observed, mainly members of Actinobacteria and Chloroflexi, whereas the abundance of Acidobacteria and Deltaproteobacteria decreased [210]. Nelson et al. [211] reported enormous changes in microbial composition in forest soils after wildfires which occur more often due to climate change. As expected, an overall decrease in the abundance and biodiversity of bacterial and fungal communities was observed one year after the fire. Both the carbon and nitrogen cycle were found to be impaired not only by the loss of microbial taxa involved in geochemical cycles, (e.g., no expression of *nifA* genes in tested soils was detected), but also by the activity of viruses. The presence of plant pathogens has also a substantial effect on the microbiome (Figure 1). In *Gossypium hirsutum* L. (cotton) infected with the pathogenic fungus *Verticillium,* the abundance of arbuscular mycorrhiza fungi and plant growth-promoting bacteria (PGPB) was lowered [212]. A study on strawberries (*Fragaria x ananassa* Duchesne) infected with *Verticillium dahliae* and *Macrophomina phaseolina* showed that plants without symptoms of infection had a higher abundance of PGPB than plants with visible symptoms of infection. The microbiome of healthy plants includes more bacteria antagonistic or competitive towards pathogens [213]. Moreover, herbivores can shape the plant microbiome. Kong et al. [214] showed that whitefly infestation of *Capsicum annuum* L. (pepper) changed the overall microbiome composition. *Pseudomonas* spp. that are recruited by the plant to the rhizosphere microbiome increased the mortality of whitefly.

A particular part of the microbiome is the core microbiome which consists of those species of microbes that regularly and ubiquitously appear in the microbiomes of particular plant species. The concept of the core microbiome was first coined by a scientist involved in the Human Microbiome Project, with the goal to identify microbial taxa and/or genes that are shared by all or most humans [215]. Microbial taxa that are commonly found in a number of environments or host types can be assigned to a core microbiome. The most common approach to verifying whether a species belong to the core microbiome is to determine microbial groups that are shared among two or more microbiomes of a particular host in various environments [216]. For example, analysis of the *B. napus* rhizosphere microbiome grown in different conditions, i.e., the level of fertilization and the level of plant density, revealed that the core root microbiome of this plant is composed of microbes belonging to genera *Streptomyces*, *Cryocola*, *Arthrobacter*, *Flavobacterium*, *Janthinobacterium*, *Serratia*, *Kaistobacter*, *Pseudomonas*, *Pedobacter*, *Agrobacterium*, *Burkholderia*, *Acidovorax, Erwinia*, and *Stentrophomonas* [217]. Analysis of the composition of the microbiome of various plant species including *A. thaliana*, rice, sugarcane, grapevine, barley, and soybean has revealed that the core microbiome included microbes belonging to *Pseudomonas*, *Agrobacterium*, *Methylobacterium*, *Sphingomonas*, *Erwinia*, *Cladosporium*, *Conithyrium*, *Resinicium*, and *Fusarium* [177]. A special part of the core microbiome is a group of microorganisms called “hub microorganisms”. Those microorganisms strongly shape the composition of the microbiome through biotic interactions with host plants and other microbes. Microorganisms belonging to the hub species are called keystone species since they serve as mediators between the plant and members of the plant microbiome. Through the hub species, the host plant can selectively affect the composition of the associated microbiome. Removal of keystone species can result in the loss of interaction and a disturbance in the whole microbiome [218]. The analysis of the phyllosphere microbiome of *A. thaliana* showed that plant-parasitic oomycetes *Albugo laibachii* is a hub species that strongly affects the whole microbial community. As a consequence of infection with *A. laibachii,* high divergence between the composition of the microbiome of control and of infected plants was observed. Moreover, less variability among microbiomes of infected plants was shown [219]. In a study on corn (*Zea mays* L.) hub species, the elimination of *Enterobacter cloaceae* from inoculum containing seven microbes resulted in a loss of a few other microbes from the microbiome. Removal of other bacteria from this system did not significantly change the microbial community which suggests that *E. cloaceae* functions as a keystone species [220]. 

A far wider concept than microbiome is the concept of holobiont which was introduced in 1991 [221]. Currently, a holobiont is defined as an organism composed of the plant host and of all the microorganisms that are associated with that particular plant. Natural selection between the plant and microbes supports the system and its stability throughout the evolution of a holobiont. In a holobiont, intricate networks of interactions between microorganisms and plant host are observed (as reviewed in [177,218,222,223]). All the genes present in the holobiont, i.e., plant genes and genes in the microbiome, constitute the hologenome [224,225]. The concept of a hologenome suggests that plants’ adaptability to the environment is determined not only by plant genes but also by genes of microorganisms. Hologenomes are responsible for shaping the phenotype of holobionts in response to a particular set of environmental conditions [226].

## 4. Plant Immune System in Plant–PGPM Interactions

The plant immune system plays a key role in plant–microorganism interactions. It is crucial not only for controlling pathogenic microorganisms but also for balancing the homeostasis of the microbiome and for overseeing commensal microbes [227]. The prominent role in plant–PGPM interactions play patterns recognizing receptors (PRRs), which recognize conserved microorganisms-specific molecules referred to as pathogen-/microbe-associated molecular patterns (P/MAMPs), such as flagellin, lipopolysaccharides, antibiotics, and VOCs [228]. PRRs are transmembrane multimeric protein complexes located at the plasma membranes present in all plant organs and tissues. Plant PRRs are either surface-localized receptor kinases that contain ligand-binding ectodomain and intracellular kinase domain or receptor-like proteins that do not have any intracellular signaling domain. PRRs contain various ligand-binding ectodomains that allow for the recognition of a wide range of P/MAMPs [228] and activate pattern-triggered immunity (PTI) [229]. Activation of PTI via MAMPs inhibits intensive proliferation of most microorganisms via synthesis and secretion of low-molecular-weight compounds, e.g., phytoanticipins and proteins, e.g., defensins. Moreover, plants synthesize cuticles which lead to the thickening of the cell wall [227]. Some pathogens secrete effector molecules, which disturb PTI functioning and thus allow for the infection of the plant [222]. Effector-triggered immunity (ETI) is activated by recognition of pathogen effector proteins via intracellular receptors R proteins encoded by resistance genes (R genes) [230,231] and via nucleotide-binding leucine-rich repeat receptors (NLR) located in the cytoplasm [229]. ETI leads to the overproduction of reactive oxygen species (ROS) and ion fluxes. As a consequence, hypersensitive response (HR) is activated which leads to apoptosis of infected plant cells that restrict the spread of infection [232].

Much less is known about the action of the plant immune system in the context of commensal microorganisms; however, there is some evidence that the plant immune system is crucial for microbiome assembly. Some strains of bacteria are able to modulate plant receptors, transcription factors, and molecules involved in the functioning of an immune system which allows for the colonization of plant tissues by other symbiotic microbes [233]. Moreover, some mechanisms used by the members of the microbiome to evade or suppress the plant immune system were also described. For example, in some symbiotic microorganisms, MAMP variants that do not activate plant immune response via PRRs have evolved. In addition, some commensal fungi are able to convert chitin into chitosan via deacetylation which induces a weaker immune response. MAMPs could be also degraded or sequestered by microbial proteases and other enzymes in order to evade recognition by PRR, as reviewed in [222]. It was also suggested that plants are able to actively ignore the presence of microbial commensals [234]. In *A. thaliana,* outer layers of roots low expression of PRRs and a lack of immune response in presence of pathogen- and commensal-derived MAMPs were reported. Neighboring cells harbor a high number of PRRs and show a rapid MAMP-triggered response [235].

## 5. Mechanisms Underneath PGPM–Plant Interactions

Plant growth-promoting microorganisms affect various aspects of plant growth and development. PGPM enhances the germination ratio [18,23,236], increases the elongation growth of the shoot and root [46,48,237], increases the biomass production [20,26,37], accelerates flowering [56,73], and increases the photosynthesis rate [27,42]. The examples of the mechanisms of action of plant growth-promoting fungi (PGPF) and bacteria (PGPB) are presented in Table 2 and Table 3, respectively.

Several mechanisms underneath the promotion of plant growth and development by microorganisms are employed by both bacteria and fungi for example degradation of ethylene via ACC deaminase, production of phytohormones, and solubilization of various soil compounds to increase the bioavailability of nutrients (Table 2 and Table 3). For sure the mechanism of greatest importance is the fixation of atmospheric nitrogen via nitrogenase, a mechanism that is specific to some specialized groups of prokaryotes (as reviewed in [271,272]). On the other hand, the fungi-specific mechanisms that allow for the promotion of plant growth and development includes the production of hydrophobins, swollenins, and peptaibols (please see the section *Trichoderma*-plant interaction—a case study, for details).

### 5.1. Plant Antioxidant Defence System

One of the best-known mechanisms to improve plant growth and development by PGPM is the modification of the level of antioxidants including antioxidative enzymes, e.g., superoxide dismutase (SOD), ascorbate peroxidase (APX), peroxidase (POD), catalase (CAT), glutathione reductase (GR), and non-enzymatic antioxidants, e.g., proline, glutathione (GSH), ascorbic acid, carotenoids, and phenolics [25,273,274,275,276,277]. Islam et al. [278] demonstrated that inoculation of *Vigna radiata* (L.) R. Wilczek with *Bacillus cereus* Pb25 increased dry biomass and yield in salt stress conditions. Salt-induced oxidative damage was reduced by enhancing the activity of plant POD, SOD, and CAT and by increasing proline content in plants. Inoculation of *O. sativa* with *Bacillus pumilus* increased the activity of rice catalase and superoxide dismutase in salt stress conditions. Moreover, inoculation of rice with *B. pumilus* promoted the synthesis of photosynthetic pigments and proline [275]. Accumulation of phenols and proline was observed in cucumber inoculated with arbuscular mycorrhizal fungi in salt conditions [279]. A potato co-inoculated with *T. viride* and plant pathogen *Alternaria solani* showed improved redox homeostasis via increased activity of CAT and SOD, and enhanced concentration of free phenolics. Moreover, co-inoculation with *T. viride* and *A. solani* resulted in increased H_2_O_2_ production which induced the expression of plant defense genes [274]. Chen et al. [25] reported increased salt stress tolerance in maize after inoculation with *B. amyloliquefaciens* SQR9. Inoculated plants showed a reduced level of Na^+^, a higher glutathione content, a higher concentration of soluble sugars, and enhanced activities of peroxidase and catalase. *B. amyloliquefaciens* enhanced chlorophyll content and promoted the overall growth of inoculated plants in comparison to control plants. In addition to the well-known elements of the antioxidant system, there are also other proteins exhibiting antioxidant activity, including small cysteine-rich proteins metallothioneins (MTs). MTs act as direct antioxidants since the reduced thiol groups (-SH) can be oxidized by ROS. MTs also serve as a donor of zinc and copper to other antioxidative enzymes [280,281]. Inoculation of rapeseed with fungal strains isolated from forest soil showed varied expressions of *B. napus* metallothioneins (*BnMT1*-*BnMT3*). *L. laccata* inoculated plants showed significant upregulation of *BnMT2* expression with a decrease in *BnMT3* transcripts [43].

### 5.2. Phytohormones

Some bacteria are able to synthesize and secrete phytohormones and thus regulate plant growth and development. For example, *B. subtills* synthesizes auxins and gibberellins [282], and *A. brasilense* [283,284], *Peanibacillus polymyxa* [285], and *P. fluorescens* [286] produce cytokinins. Inoculation of *S. tuberosum* damaged by insect attack (beetle *Leptinotarsa decemlineata*) with *B. subtilis* 26D led to increased concentration of zeatin-riboside but not of abscisic acid (ABA) and indole acetic acid (IAA). The inoculation of potatoes with *B. subtilis* increased the mass of roots [287]. In the culture of *Bacillus aryabhattai* abscisic acid, indole acetic acid, cytokinins, and gibberellic acids were detected. Soybean inoculated with these bacteria produced more IAA, jasmonic acid, and some gibberellic acids. Moreover, inoculated plants displayed increased tolerance to heat stress possibly due to the ABA-induced closure of stomata [288].

Although different stressors affect plant organisms in various ways, most of them lead to increased ethylene production in plants. Weak stress factors can cause small overproduction of ethylene which leads to the activation of plant stress-related genes. Long periods of stress and severe stressors cause a high level of ethylene production, which might lead to senescence, chlorosis, and organ abscission [136]. Some PGPM are able to lower the level of ethylene through secretion of ACC deaminase, i.e., the enzyme that breaks down ethylene precursor 1-aminocyclopropane-1-carboxylate (ACC). Inoculation of plants with microbes producing ACC deaminase effectively increased the plant resistance to stress caused by fungal pathogens [289], nematodes [290], and several abiotic stresses such as flooding [291], drought [135], salination [133], heavy metals [292], and toxic contaminates [134]. For example, inoculation of pearl millet seed with ACC deaminase-producing *B. amyloliquefaciens* increased plant growth in drought stress via increasing the level of enzymatic and non-enzymatic antioxidants [272]. Isolated from *Brassica rapa* L. rhizosphere bacteria belonging to *Pseudomonas* sp. improved biomass and yield of *B. rapa*. The positive effect of analyzed strains on *B. rapa* is possibly due to the production of IAA, ACC deaminase, and siderophores [95].

### 5.3. Availability of Micro- and Macronutrients

In most terrestrial ecosystems, nitrogen is the major nutrient limiting plant growth. PGPMs might increase the pool of nitrogen available for plants thorough various mechanisms. It is estimated that in nature more than 60% of fixed nitrogen is a result of biological nitrogen fixation [272]. A well-known example is the *Rhizobia* present in root and stem nodules of legumes that is able to reduce N_2_ to ammonia. There are also other symbiotic, plant-endophytic, and free-living bacteria able to fix molecular nitrogen, including bacteria belonging to *Frankia*, *Cyanobacteria*, *Azotobacter*, *Bacillus*, and *Azospirillum,* as reviewed in [293]. The Co-inoculation of beans with *Rhizobium phaseoli* and bacteria belonging to *Bacillus* and *Pseudomonas* improved plant growth by enhancing the total content of nitrogen in plant tissues more efficiently than inoculation with *R. phaseoli.* Only [127]. Hungria et al. [130] reported that the co-inoculation of soybean seeds with *Azospirillum* and *Bradyrhizobium* significantly enhances the yield without any input of nitrogen fertilizers. Several lines of evidence showed that rhizobia are susceptible to drought-stress and the efficiency of N_2_-fixation dramatically declines in low-water conditions [294]. For example, co-inoculation of a common bean with *Rhizobium tropici* and two strains of *P. polymyxa* more effectively increased nitrogen content and promoted plant growth than inoculation with *Rhizobium* only especially in drought conditions [295].

Phosphorus is also considered as element limiting plant growth in most ecosystems. Although phosphorus is an abundant element in ecosystems, most of it is not bioavailable [296]. There is a group of microbes, phosphate solubilizing microorganisms (PSM), that are able to increase the available fraction of phosphorus for plants via solubilization and mineralization mediated by secretion of organic acids, phosphatases, protons, and exopolysaccharides [297]. Red clover (*Trifolium pratense* L.) inoculated with phosphate solubilizing fungi *Penicillium albidum* showed a significant increase in root biomass. In soil inoculated with fungi, the phosphatase activity was 1.5-fold higher than in the non-inoculated soil [129]. In soil inoculated with *Burkholderia* sp., *Gluconobacter* sp., and *Pseudomonas striata,* higher activity of dehydrogenase and phosphatase and a higher level of available P were detected. *Vigna unguiculata* (L.) Walp. grown in inoculated soil had noticeably higher biomass and yield than plants grown in non-inoculated soil. Moreover, tested microorganisms enhance uptake not only of phosphorus but also of nitrogen [298]. Inoculation of *T. aestivum* with *Serratia marcescens* enhanced plant growth and nutrient uptake (P, N, and K) in low temperatures. The ability of tested bacteria to solubilize P decreased at low temperatures [299].

Soil minerals, such as feldspars and micas, are the most common form of potassium in soils. Up to 90–98% of soil potassium is present in a form unavailable for plants [300]. By secretion of organic acids and capsular polysaccharides, some PGPM are able to solubilize potassium rocks, e.g., bacteria belonging to *Acidothiobacillus*, *Bacillus*, *Pseudomonas*, *Burkholderia*, and *Peanibacillus* [301]. Ali et al. [302] reported that inoculation of potatoes with K-solubilizing *B. cereus* resulted in significantly improved plant growth and yield. Moreover, the content of K in potato tubers and the content of N, P, and K in leaves was higher in comparison to control plants. Inoculation of ryegrass with *Mesorhizobium* sp., *Peanibacillus* sp., and *Arthrobacter* sp. isolated from canola rhizosphere improved biomass and yield. The content of available K in soil was much higher and resulted in increased K content in plants [303]. Basak and Biswas [304] demonstrated that treatment of waste mica with *Bacillus mucilaginosus* led to the transformation of K forms into water-soluble forms. This had a positive effect on K uptake and biomass of sudan grass (*Sorghum vulgare* Pers.). Similar effects were observed by Raji and Thangavelu [305], who analyzed the effect of inoculation of tomato (*Lycopersicon esculentum* L.) grown in Alfisol and Vertisol soils with *B. subtilis*, *B. cereus*, *Bacillus licheniformis,* and *Burkholderia cenocepacia*. Inoculated plants showed higher K content in tissues and improved growth.

The deficiency of microelements, including copper (Cu), iron (Fe), zinc (Zn), and manganese (Mn), is also a prominent factor that negatively affects plant health. In calcareous soils (widespread in arid and semiarid regions of the world), the high content of calcium carbonate which acts as a buffer and maintains a pH above 7.5, is correlated with decreased bioavailability of Fe, Zn, and Mn [306]. Plants possess mechanisms allowing them to increase amounts of bioavailable microelements in soils. For example, to facilitate Fe acquisition, plants secrete siderophores, organic acids, and flavonoids [307], whereas the amount of bioavailable Zn is increased by the secretion of organic acids, such as propionic acid, formic acid, lactic acid, citric acid, succinic acid, malic acid, oxalic acid, and gluconic acid as well as by secretion of siderophores, as reviewed in [308]. A tomato grown in hydroponic culture with the addition of soil minerals in Cu-deficient conditions showed increased biomass and Cu uptake after inoculation with *Trichoderma harzianum* SQR-T037 in comparison to non-inoculated plants. Interestingly, inoculation of a tomato with *T. harzianum* SQR-T037 grown in an Fe-deficient hydroponic culture with the addition of solid mineral increased the Fe content in plant tissue, but the biomass of seedling was unaffected. In addition, inoculation of tomatoes grown in Zn-deficient hydroponic conditions did not increase the biomass and the concentration of Zn in plant tissues was decreased. Those observations suggest that in element-deficient conditions fungi compete with plants for nutrients [309]. Rana et al. [128] showed enhanced yield and increased concentrations of Fe, Zn, Cu, and Mn by 13–16% in rice grains after inoculation with *Brevundimonas diminuta*, *Ochrobactrum anthropic*, and *Providencia* sp. Wheat inoculation with *Providencia* sp. significantly increased yield and the content of Fe and Cu in grains was 45% higher than in control plants. Singh et al. [310] reported that endophytic bacteria isolated from wheat, such as *B. subtilis*, *Arthrobacter* sp., *A. sulfonivorans*, and *Enterobacter hirae* exhibiting plant growth-promoting traits, enhanced Fe and Zn fortification as well as yield and dry/fresh weight in pot experiment and field conditions. Moreover, a decrease in phytic acid was observed in grains of wheat plants inoculated with endophytes. A field study on common bean and wheat fortification showed that inoculation with *A. brasilense* and *T. harzianum* significantly enhanced micronutrients, i.e., Fe, Mn, and Zn content in both tested plants [311]. Enhanced content of selenium was observed in lettuce inoculated with bacteria *Bacillus* sp., *Klebsiella* sp., *Acinetobacter* sp., and with fungus *Rhizophagus intraradices* in drought stress conditions. Results showed that plants inoculated with bacteria showed higher biomass production in comparison to plants inoculated with fungus, and *Klebsiella* sp. was the most effective in the induction of Se accumulation in lettuce. Moreover, tested microbes increased drought stress resistance, the chlorophyll and carotenoid content, and enhanced the level of antioxidant enzymes [312]. The ability of some PGPM to increase the content of macro- and micro-elements in different parts of plants is also important for human nutrition. Microelements deficiency (especially zinc and iron) is widespread all over the world. Biofortification, i.e., approaches to enhance the nutritional value of crops, with microelements in edible parts of plants, is the most promising approach to fight against microelements deficiency, as reviewed in [313,314,315]. Therefore, PGPM seems to be of crucial importance for both food quantity, i.e., enhanced plant yield and food quality, i.e., edible parts of plants with high content of minerals.

### 5.4. Direct Interactions of PGPM with Plant Pathogens

PGPM might directly interact with various plant pathogens. Through various mechanisms, including the production and secretion of antimicrobial metabolites, antagonisms, mycoparasitisms, and competing for niche PGPM can enhance plant biotic stress resistance. Cabrefiga et al. [316] reported an antagonistic interaction between *P. fluorescens* EPS62e and *Erwinia amylovora*, bacteria causing fire blight disease on pear trees (*Pyrus* sp.). *P. fluorescens* EPS62e did not produce antibiotics and required cell-to-cell contact with the pathogen in order to inhibit their growth on pear flowers and fruits. Interestingly, antagonistic activity was not shown when bacteria were grown in an Fe-rich medium, which suggested that the production of siderophores is responsible for the *P. fluorescens* EPS62e ability to inhibit the growth of *E. amylovora*. Inoculation of plants with antibiotic-producing microorganisms might lead to the suppression of various plant diseases. From the roots and rhizosphere soil of cucumber grown in soil inoculated with biocontrol agent *B. subtilis,* antibiotics surfactin and iturin A were extracted [317]. Bais et al. [318] showed that the biocontrol ability of *B. subtilis* against *Pseudomonas syringae* is tightly linked with the production of surfactin involved in biofilm formation on *A. thaliana* roots. *B. subtilis* mutant with a deletion in the surfactin synthase gene was unable to form biofilm and was ineffective in protection against *P. syringae* attack. Rhizospheric *S. plymuthica* HRO-C48 isolated from the rhizosphere of rapeseed produces antibiotic pyrrolnitrin and can protect plants against Verticillium wilt [319]. Kurze et al. [320] demonstrated that *S. plymuthica* HRO-C48 protects strawberries against fungal pathogens *V. dahliae* and *Phytophtora cactorum*. Moreover, the inoculation of strawberries with *S. plymuthica* promoted plant growth and improved yield. Inoculation of wheat seedlings with *Trichoderma* sp. resulted in an increase in plant resistance markers when plants were infected with *Fusarium* spp. Moreover, a decrease in IAA was observed. Biocontrol activity of *Trichoderma* was connected with the secretion of lytic enzymes and fungal elicitors as well as mycoparasitism [49]. An interesting observation was made by Chen et al. [321] who showed that *Pseudomonas piscium* isolated from wheat is able to alter histone acetylation in pathogenic *Fusarium gramineareum* and, therefore, reduce the fungus level of virulence and mycotoxin production by the fungus. An identified compound secreted by *P. piscium,* i.e., phenazine-1-carboxamide, disturbs the activity of fungal histone acetyltransferase FgGcn5, responsible for the regulation of gene expression involved not only in virulence and the growth of the mycelium, but also in asexual and sexual reproduction and stress response.

### 5.5. Induction of the Plant Resistance by Microbial Elicitors

Although the use of living microorganisms is a potent tool in the development of sustainable agriculture, it has also numerous constraints due to legal regulations [322]. As an alternative to the living microbes and cell wall polymers (CWP) of bacteria, fungi can be employed as elicitors [323,324,325]. Elicitors trigger the immune response of the plant via numerous mechanisms including accumulation of lignin, antimicrobial enzymes, e.g., chitinases, glucanases, phytoalexins, and proteins related to the response to the presence of pathogens, guaiacol, and ribonuclease [326]. One of the efficient elicitors is chitin and its deacetylated derivative chitosan (N-acetylglucosamine subunits are linked by (1 → 4) -β bonds). For example, chitosan led to an increase in the systemic resistance in tomatoes and an increase in the plant resistance towards *Alternaria solani* and *Xanthomonas vesicatoria* [327]. Treatment of *Psammosilene tunicoides* with chitosan led to an increased expression of genes encoding antioxidant enzymes and transcription factors controlling stress-response genes. Moreover, the content of secondary metabolites terpenoid saponins increased [328]. The cost-effective and efficient source of elicitors are fungi belonging both to Ascomycota and Basidiomycota. Research by Nowak et al. [326] showed that (1 → 3) -α-D-glucooligosaccharides (GOS) obtained by hydrolysis of (1→3)- α-D-glucan from *Laetiporus sulphureus* induced the growth of wheat seedlings. Moreover, GOS caused the increase in the activity of CAT and APX, in the activity of chitinase, and higher activity of enzymes activating phenylpropanoid-producing pathways. Laminarin, a polysaccharide, consists of β-(1-3)-glucan with β-(1-6)-linkages of 20–25 units isolated from brown algae, which is an example of elicitor belonging to β-glucans. Treatment of grapevine-cultured cells with laminarin led to calcium influx, an oxidative burst, and the induction of pathogen-related genes. Laminarin by the induction of plant resistance indirectly contributes to the reduction of the growth of *B. cinerea* and *Plasmopara viticola* on grapevine plants [329].

## 6. The Stringent Response in Plant–Microorganism Interactions

Among several plant mechanisms regulating growth, development, and the response to environmental factors, the stringent response is of particular interest. The stringent response was first discovered in *Escherichia coli* in response to amino acid starvation [330]. The hallmark of the stringent response is the accumulation of atypical regulatory nucleotides guanosine tetra- (ppGpp) and pentaphosphates (pppGpp) called alarmones that are responsible for pleiotropic adaptation to nutrient deficiency and stress factors [331,332]. Moreover, the bacterial stringent response through regulation of quorum sensing indirectly affects the formation of microcolonies and the development and functioning of biofilm [333]. Alarmones are synthesized from ATP/GTP or GDP by enzymes possessing active synthetase domain (SYNTH) and are hydrolyzed to GTP/GDP and pyrophosphate by enzymes containing active hydrolytic domain (HD). Gram-negative bacteria usually possess two separate enzymes, i.e., alarmones synthetase RelA and alarmones hydrolase SpoT. Gram-positive bacteria usually possess one bifunctional Rel protein [334,335]. RelA and SpoT belong to the long RSH (RelA/SpoT homologue), i.e., enzymes possessing HD and SYNTH domains. In bacteria, there are also short RSH, i.e., enzymes containing either SYNTH domain, small alarmone synthases (SAS) or HD domain, or small alarmone hydrolases (SAH), whereas in animals, to date only small alarmone hydrolases were identified (Mesh1—metazoan SpoT homologue) [336]. Alarmones regulate transcription, translation, and DNA replication, and trigger metabolical and physiological changes in response to unfavorable environmental conditions. Upon accumulation of alarmones, bacteria change their lifestyle from growth and proliferation to survival mode [335]. Sanchez-Vazquez et al. [337] demonstrated that in *E. coli,* the elevated (p)ppGpp level affected the expression of 757 genes five minutes after the induction of the stringent response and after another five minutes the expression of 1 224 genes was affected. Activation of the stringent response can be triggered by a deficiency of amino acids [330], fatty acids [338,339], iron [340], carbon [341], nitrogen [342], phosphorus [343], and by other types of stress, e.g., increased temperature [344,345], cell wall antibiotics, ethanol and acid treatments, superoxide stress [346], and alkaline shock [347].

The presence of (p)ppGpp in photosynthetic *Eucaryota* was confirmed in the algae *Chlamydomonas reinharditi* [348]. In higher plants, *RSH* genes were identified for the first time in *A. thaliana* [349] and subsequently in other plant species, e.g., *Nicotiana tabacum* L. [350], rice [351], *Ipomoea nil* L. Roth [352], *Sueda japonica* Forssk. ex J.F. Gmel. [353], pepper [354], and non-vascular plants, e.g., *Physcomitrella patens* (Hedw.) Bruch and Schimp [355]. Plant RSH proteins are divided into three groups, i.e., RSH1, RSH2/3, and CRSH. All identified plant RSH proteins belong to the long RSH, i.e., possess both HD and SYNTH domains. In model plants, the *A. thaliana* SYNTH domain of RSH1 (AtRSH1) is Inactive due to the substitution of conserved glycine residue required for its activity. AtCRSH does not possess a functional hydrolase domain and in AtRSH2/3, both SYNTH and HD domains are active [356]. In addition to HD and SYNTH domains, RSH possess chloroplast transit peptide [357], TGS (RSH1, RSH2/3), and ACT domains (RSH1) which were proposed to act as regulatory- or ligand-binding domains [358]. CRSH are the only proteins involved in alarmones metabolism that possess two EF hand motifs and are activated by Ca^2+^ ions [359]. The domain structure of RSH proteins is highly conserved across plant species. It is now widely accepted that in plants, the place of alarmones action are chloroplasts [360,361,362,363]. Alarmones act as regulators of plant development and growth, i.e., (p)ppGpp coordinate micro- and macro-elements redistribution during senescent [364]. They modulate the level of phytohormones [365], lipids [366,367], and secondary metabolites [368] in chloroplasts. Moreover, alarmones promote the replication of plastidial DNA [364]. Plant *RSH* genes are differently expressed in presence of biotic and abiotic stress factors, which suggest that alarmones contribute to plant response in a number of stress factors, i.e., oxidative stress [369], nitrogen starvation [369,370], wounding, salination, drought, UV radiation, heat shock, heavy metals, and abrupt change from light to darkness [371]. Masuda et al. [372] showed that RSH probably play a role in plant reproduction, as the *AtCRSH* knockdown mutant produced smaller siliques and a lower number of seeds. An interesting observation was made by Ono et al. [373], who demonstrated an increased (p)ppGpp concentration in chloroplast upon a light-to-dark transition. The alarmones accumulation was due to higher activity of CRSH caused by elevated levels of Ca^2+^ in chloroplasts. As a consequence, the expression of plastidial genes is adapted to darkness. Interestingly, it was demonstrated that the accumulation of alarmones in plants leads to a decreased expression of genes involved in the defense system, which can lead to higher susceptibility to infections [365].

Plants are probably able to manipulate the level of alarmones synthesis in members of their own microbiome but also in pathogenic bacteria. Through modulation of (p)ppGpp production in bacteria, plants may be able to decrease virulence and inhibit the growth of pathogens. Nowicki et al. [374] demonstrated the activation of stringent response in *E. coli* cells by plant secondary metabolites isothiocyanates (ITC). ITC-induced stringent response in *E. coli* led to growth inhibition, disturbed transcription, and DNA replication. The induction of the *E. coli* stringent response may be the result of a direct interaction of ITC with cellular proteins. That idea is plausible since sulforaphane (one of the ITCs) inhibits the growth of numerous bacteria and recently potential target proteins of ITC were identified [375]. Mwita et al. [376] showed that the expression of *SasA* (short alarmone synthase) of PGPB *Bacillus atrophaeus* UCMB-5137 is considerably upregulated by maize root exudates. Further implications of observation on these bacteria metabolisms need, however, further evaluation. On the other hand, inoculation of plants with PGPB can affect plant *RSH* gene expression; however, the data are scarce. Dąbrowska et al. [377] demonstrated that *B. napus* inoculated with *S. plymuthica* and *Serratia liquefaciens* exhibited elevated mRNA levels of *BnRSH1* in cotyledons and roots, whereas inoculation with *Massilia timonae* increased *BnRSH1* expression only in roots. Moreover, *S. plymuthica* seemed to affect also the expression of *BnRSH2* and *BnRSH3* in cotyledons and roots. The relative transcript level of *BnCRSH* was elevated in cotyledons in presence of *S. plymuthica* and *S. liquefaciens*. Inoculation of canola grown in salt-stress conditions with endophytic *Pseudomonas stutzeri* ISE12 significantly increased mRNA levels of *BnRSH1* and *BnRSH3* in roots in comparison to non-inoculated plants grown in salt stress [34]. Moreover, Givens et al. [350] observed a 10-fold increase in RSH2 protein level in *N. tabacum* after infecting the plant with the bacterial pathogen *Erwinia carotovora*.

Microbes might directly and/or indirectly activate the stringent response in other microorganisms. A study on the effect of the pathogenic fungus *Rhizoctonia solani* on the rhizosphere microbiome of sugar beet (*Beta vulgaris* L.) showed that in several rhizobacteria, the expression of genes involved in (p)ppGpp metabolism is upregulated. It is not clear whether the activation of the stringent response in bacteria present in the rhizosphere is triggered directly by oxalic and phenylacetic acids secreted by *R. solani* or indirectly by signaling molecules in root exudates [378]. Interestingly, the *relA* and *relA*/*spoT* mutants of *Pseudomonas* sp. DF41 and *Pseudomonas chlororaphis* PA23, showed increased antifungal activity against *Sclerotinia sclerotiorum*. All mutants produced an increased level of antifungal antibiotic pyrrolnitrin, lipase, and protease in comparison to wild-type bacteria. The lack of (p)ppGpp led also to reduced transcription of *rpoS* [379,380]. Selin et al. [381] showed that expression of *rsmZ*, *rsmE,* and *rsmA,* i.e., elements involved in the regulation of several processes such as virulence, motility, and biocontrol abilities, was regulated via the stringent response in *P. fluorescens*, a PGPB inhibiting the growth of a number of pathogenic fungi including *S. sclerotiorum*. Takeuchi et al. [382] reported that a mutant of *P. fluorescens* CHA0 lacking the ability to synthesize alarmones, produced significantly fewer antibiotics and had lower biocontrol activity against *P. ultimum*. Moreover, the ability of the tested mutant to colonize cucumber roots, both in the presence and absence of *P. ultimum*, was reduced possibly due to impaired motility. The stringent response was found to strongly influence the production of antibiotics also in *Streptomyces* which are common members of the plant microbiome. Ochi [383] reported that *rel* mutant of *Streptomyces antibioticus* showed induction of phenoxazinone synthase, an enzyme involved in the production of actinomycin. Interestingly, this mutation did not affect the activity of another enzyme participating in the biosynthesis of actinomycin, i.e., kynurenine formamidase. Moreover, *RSH* genes regulated the morphological and physiological differentiation of *Streptomyces clavuligerus*; the lack of (p)ppGpp affected spore formation [384].

Recent studies showed that the stringent response may play a crucial role in the interaction between legumes and rhizobia. At the beginning of the interaction, legumes produce antimicrobial compounds that lead to nutritional, osmotic, and oxidative stress in rhizobia [385,386,387]. Based on the literature, it might be hypothesized that in order to survive these unfavorable conditions, rhizobia activates the stringent response. Soybean inoculated with the rsh knockout *Bradyrhizobium diazoefficiens* formed smaller nodules than those present in plants inoculated with wild-type *B. diazoefficiens*. Moreover, the biomass of plants inoculated with mutant bacteria was smaller in comparison to wild-type bacteria, but still higher than the biomass of non-inoculated plants. Those results suggest that the lack of alarmone signaling altered nodulation and, as a consequence, decreased N_2_ fixation. Interestingly, in plants co-inoculated with wild-type bacteria and the *rsh* mutant in equal proportion, only 26% of nodules were infected by mutant bacteria. This observation strongly suggests that the stringent response is crucial to win the competition for a niche with other rhizobia [388]. In the early phase of nodulation, establishing a symbiotic relationship between rhizobia and legumes requires plant-bacteria signaling that allows the recognition of bacteria by the host. It was shown that *relA* mutant of *Sinorhizobium meliloti* failed to form nodules on *M. sativa* due to disturbed stringent response, but remarkably the nodulation of *Medicago truncatula* Gaertn. was successful. The N_2_ fixation capacity of mutant bacteria was reduced in comparison to the wild type. It is not clear why there is a difference in nodulation between those two *Medicago* species; however, it might be hypothesized that different plants influenced bacterial stringent response at different stages of invasion [389]. Another study also found that *S. meliloti* mutant unable to synthesize (p)ppGpp did not establish a symbiotic relationship with *M. sativa*. The *relA* mutant of *S. meliloti* produced more succinoglycan, an exopolysaccharide needed for colonization of the host, than the wild-type bacteria [390]. The inactivation of *relA* in *Rhizobium etli* caused nodules on *P. vulgaris* form, but the level of nitrogen fixation was significantly reduced. Mutants showed significantly lower expression of *raiI* and *cinI* genes which encode regulators of quorum sensing in rhizobia [391]. Calderón-Flores et al. [392] reported that in *P. vulgaris* inoculated with *R. etli,* the rsh mutant nodulation and nitrogen fixation were disturbed. Qiu et al. [393] demonstrated that the addition of water-soluble humic materials into inoculum containing *Sinorhizobium fredii* significantly downregulated *RSH* expression, increased survivability of bacteria in soil, and promoted rhizoplane colonization of *Glycine max* (L.) Merr.

The stringent response is also a significant mechanism contributing to the virulence of various plant pathogens. A study on *E. amylovora* showed that cells of *relA* and *relA*/*spoT* mutants were significantly longer both in nutrient-rich and nutrient-limited conditions in comparison to the wild type. Moreover, it was demonstrated that the proliferation rate of *relA*/*spoT* mutant in pear fruits was 1000 times slower than the proliferation rate of the wild type. In minimal medium double, the *relA*/*spoT* mutant was unable to grow. Small-sized cells are more resistant to stress, and bigger *relA*/*spoT* knockouts are unable to survive on plant surfaces. It seems that (p)ppGpp are important regulators of cell growth in *E. amylovora* during plant infection [394]. A similar observation was made for *P. syringae*, one of the most common plant pathogens. *P. syringae* single (*relA*) and double (*relA*/*spoT*) knockout mutants grown on nutrient-rich medium were slightly bigger in comparison to the wild-type bacteria. An in vivo study demonstrated that *relA*/*spoT* mutants, even though they had bigger cells, were unable to survive on the surface of a tomato leaf. These findings suggest that the stringent response is a crucial element of plant surface colonization [395,396]. A great induction of *relA* and *spoT* genes in gram-negative bacterium *Pectobacterium atrosepticum*, able to degrade plant cell walls, was observed when bacteria were grown in high-density culture in carbon-deficient media [397,398]. Zhang et al. [399] reported that the *Xanthomonas citri* double knockout *spoT*/*relA* mutants showed a significant decrease in pathogenicity and inhibited growth in planta. Interestingly, the deletion of only the main alarmones synthase in the *X. citri relA* did not affect the virulence of the bacterium.

Several lines of evidence showed that the stringent response plays a crucial role in the adaptation of bacterial growth and metabolism to nutrient-limited conditions. The mentioned studies above show the importance of the stringent response for the establishment of plant–PGPM interactions and proper functioning of the plant microbiome. We hypothesize that the stringent response probably allows members of the microbiome to survive in unfavorable conditions during the first stages of interaction establishment. The data about the possible role of alarmones in an intricate network of interactions between plants and microorganisms, among members of microbiomes, and between PGPM and plant pathogens are rather limited. The possible crucial role of the plant and the microbial stringent response in the functioning of microbiomes is an exciting but rather overlooked research area that needs further experiments.

## 7. *Trichoderma*–Plant Interaction—A Case Study

Among several other microbes belonging to the PGPM group, fungi from the genus *Trichoderma* are of great interest. Adaptability to unfavorable environmental conditions and the ability to utilize many different substrates as nutrients determine the ubiquitous occurrence of fungi belonging to *Trichoderma* in soils. It is estimated that one gram of soil contains 10–10^3^ CFU (colony-forming unit) of fungi belonging to *Trichoderma* [400,401]. It was estimated that there are around 438 species in the genus *Trichoderma*, grouped into 10 phylogenetic lineages: Brevicompactum, Deliquescens, Harzianum, Hypocreanum, Longibrachiatum, Polysporum, Psychrophilum, Semiorbis, Stromaticum, and Viride [402].

Through interaction with plant fungi belonging to *Trichoderma* gains, a convenient niche for growth and development since root exudates are a rich source of carbon and other nutrients. The presence of fungi belonging to *Trichoderma* enhances growth and increases yield [403,404], improves uptake of nutrients by plants [405,406], and leads to a higher vigor and germination ratio of seeds [407,408]. In addition, fungi belonging to *Trichoderma* increase the level of photosynthesis [409], the level of amino acid synthesis [410], the level of transpiration [411], and the water content in tissues in drought conditions [412]. Fungi from the genus *Trichoderma* can colonize the roots of mono- [56,57,413] and di-cots [58,131,132] and when plants are grown in acidic soils [414], alkaline soils [415,416], and soils contaminated with heavy metals [417,418]. *Trichoderma* are potential symbionts of non-mycorrhizal plants belonging to *Brassicaceae* [59], *Chenopodiaceae* [302], *Caryophyllaceae* [419], *Polygonaceae* [420], and others. Recently, marine isolates of *Trichoderma* have been identified [421,422,423,424,425], which have the potential to serve as plant growth-promoting fungi for plants grown in saline soils [426]. Several mechanisms have been shown to contribute to the promotion of plant growth and development by fungi belonging to *Trichoderma*. Colonization of plants by *Trichoderma* changes host proteome [413] and secretome [427], affecting the level of synthesis of phytohormones [428] in soluble sugars [409] and phenolic compounds [173,429]. The inoculation of *A. thaliana* seedlings with *Trichoderma virens* and *Trichoderma atroviride* increased biomass production and promoted lateral root growth. Mutations in plant genes involved in auxin transport and signaling, i.e., *AUX1, BIG, EIR1*, and *AXR1* caused reduced stimulation of root growth and development by tested *Trichoderma* isolates [92]. Fungi belonging to *Trichoderma* compete with pathogens for ecological niches and nutrients, which efficiently limits the growth of pathogens. Secreting antibiotics, siderophores, a range of volatile and non-volatile metabolites (n-alkanes, cyclohexane, cyclopentane, esters, alcohols, sulfur-containing compounds, pyrane, and benzene derivatives), and through mycoparasitism fungi belonging to *Trichoderma,* protect plants against various pathogens, such as *R. solani* [430,431,432], *Rhizopus oryzae* [433], *Fusarium* spp. [434,435], *Alternaria alternate* [436], *S. sclerotiorum* [432,437], *Botrytis cinerea* [438,439], *Pythium* spp. [433], and *Ustilago maydis* [440]. Several secondary metabolites produced by fungi belonging to *Trichoderma* peptaibols seem to be of great importance for *Trichoderma* biocontrol activity. Peptaibols are amphipathic polypeptides composed of 5–10 amino acids with molecular masses between 500 and 2200 Da. These non-ribosomally synthesized polypeptides contain not only typical amino acids but also non-proteinogenic amino acids and α-aminoisobutyric acid. Peptaibols are synthesized not only by fungi belonging to *Trichoderma* but also by other soil-born fungi as well as by plant-pathogen fungi [441,442]. Several lines of evidence have confirmed that peptaibols exhibit antibacterial and antifungal properties. *Trichoderma pseudokoningii* produces trichokonin VI that induces apoptotic cell death in *F. oxysporum* [443]. Trichokonins A produced by *Trichoderma longibrachiatum* damages the cell membrane of Gram-negative pathogenic bacteria *Xanthomonas oryzae* pv. *Oryzae*, leading to a significant reduction of the pathogenicity of these bacteria [444]. The same inhibitory effect was observed for several other peptaibols produced by various *Trichoderma* species against a range of plant pathogens, e.g., *B. cinerea* [445], *Septoria tritici* [446], *A. solani*, and *R. solani* [447]. Moreover, it was also demonstrated that peptaibols can act against viruses. Luo et al. [448] showed that trichokonins isolated from *T. pseudokoningii* induces resistance of tobacco against the tobacco mosaic virus probably via induction of reactive oxygen species and phenolic compound production. Peptaibols isolated from *T. virens* might act as elicitors and induce a defense response in cucumber against pathogenic bacteria *Pseudomonas syringae* pv. *lachrymans* via up-regulation of hydroxyperoxide lyase, phenylalanine ammonia lyase, and peroxidase gene expression. In addition, *T. virens* mutant *tex1* lacking one of the non-ribosomal peptide synthetases was less effective in the inhibition of *P. syringae* pv. *lachrymans* growth [247]. It should be pointed out that high concentrations of peptaibols might have a negative impact on the growth of plants, as shown for peptaibols produces by *Trichoderma reesei* and their negative effect on *A. thaliana*. However, peptaibols at lower concentrations are still sufficient to inhibit the growth of plant pathogens with no adverse effect on plant growth [449].

Moreover, some strains of *Trichoderma* can induce ISR (induced systemic resistance) and/or SAR (systemic acquired resistance) in the host plant through the secretion of fungal elicitors. Shoresh et al. [450] demonstrated that the treatment of cucumber with *T. asperellum* T203 activated ISR via the JA/ethylene signaling pathway. Inoculated plants were more resistant to *P. syringae* than non-inoculated control plants. Another study showed that soil inoculation with *T. harzianum* enhanced tomato defense against root-knot nematode (*Meloidogyne incognita*) by SAR activation and increased ethylene synthesis [132]. Inoculation of canola with *T. harzianum* TH12 triggered SAR and ISR defense mechanisms and decreased the severity of disease symptoms caused by *S. sclerotiorum* [132]. Changes in host cells caused by inoculation with *Trichoderma* are also visible in tissues distant from the penetration site. Yedidia et al. [451] demonstrated that the cell walls of cucumber root epidermis and cortical cells after *T. harzianum* inoculation were strengthened also beyond the penetration site. The activity of plant peroxidases and chitinases was upregulated by the presence of a fungus both in roots and leaves. These findings are in agreement with the fact that microbial invasion of host plant cells induces systemic resistance mechanisms. Huang et al. [452] reported that inoculation of cucumber with *T. harzianum* SQR-T37 significantly promoted growth as well as suppressed the damping off disease caused by *R. solani*. It was shown that the main mechanism of biocontrol was mycoparasitism. Between the pathogen and *T. harzianum* SQR-T37, a direct interaction was observed. The hyphae of *T. harzianum* were densely coiled, and hooks and appressorium-like bodies were formed. As a consequence, the cell walls of *R. solani* were broken and leakage of cytoplasm was noted.

**Figure 2:**
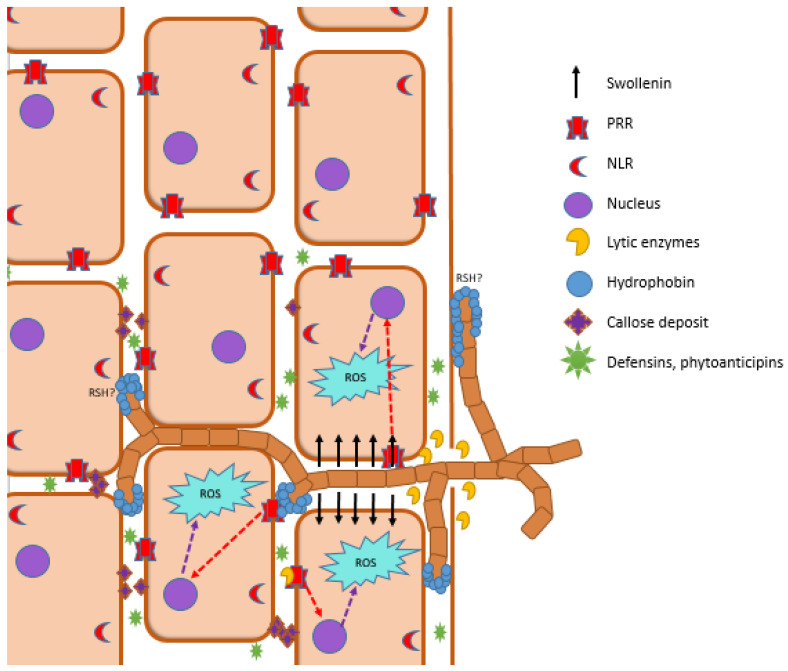
Colonization of root by a fungus belonging to *Trichoderma*. Adhesion and protection of hyphae are mediated by the layer of hydrophobins, whereas lytic enzymes enable penetration of the epidermis. Swollenins facilitate penetration of apoplast through an expansion-like effect on plant cell walls. Recognition of *Trichoderma*-derived MAMP molecules (swollenins, hydrophobins, cellulolytic enzymes, and chitin) triggers plant responses to infection, i.e., synthesis of antimicrobial compounds (defensins and phytoanticipins), synthesis of the callose wall in order to physically inhibit further penetration, and overproduction of ROS and possibly also alarmones. See text for more details.

Fungi belonging to *Trichoderma* are able to colonize plants due to the mechanisms allowing the recognition and the adhesion to the surface of the root (Figure 2). Moreover, these fungi are able to penetrate the root tissues and suppress the plant’s immune system in order to avoid strong responses from the host, as reviewed in [453,454]. The plant’s immune system recognizes *Trichoderma* MAMPs, including swolenin [250], alamethicin [455], xylanase [456], cellulases [457], and polygalacturonase [458]. Plant responses to MAMPs are quick and transitional in the early stages, they involve ion level fluctuation, overproduction of ROS, nitric oxide, and ethylene [459]. Later stages involve the production of callus wall and antifungal compounds and, as a consequence, further penetration of the plant by hyphae is stopped [429,460]. It was demonstrated that salicylic acid has a particular role in callus synthesis during the colonization of plants by fungi. In a *A. thaliana sid2* (salicylic acid induction-deficient2) mutant with a disturbed SA signalization, *T. harzianum* penetrated root vascular tissues whereas in wild-type plants, penetration of plants by fungi were restricted to outer root layers [460]. Inoculation of *Z. mays* with *T. virens* caused the reduction of plant secretome by 36%, which deprives plants of essential signaling molecules and proteins crucial for proper plant growth and development. At the same time, a fungus secretes similar compounds which do not activate the plant’s immune system [427]. The ability to colonize plant tissues by *Trichoderma* is tightly linked with the fungal capacity to tolerate secreted plants’ antimicrobial compounds [459]. An ABC transporter system is crucial for the resistance of *Trichoderma* to antifungal compounds secreted by plant pathogens. Deletion in the *Taabc2* gene in *T. atrioviride* caused the loss of the ability of the fungus to protect the tomato against *P. ultimum* and *R. solani* attacks [461]. Another mechanism exploited by fungi belonging to *Trichoderma* is directly decreasing the synthesis of the antifungal compound. For example, Masunaka et al. [462] reported that inoculation of *Lotus japonicus* L. with *Trichoderma koningi* down-regulated the production of isoflavonoid vesitol, i.e., the main phytoalexin produced by lotus species. Fungi belonging to *Trichoderma* were found proficient in the degradation of allelochemicals secreted by plants which exhibit fungitoxicity to a number of fungi [173].

Adhesion of fungi belonging to *Trichoderma* to the surface of plants is mediated by hydrophobins, i.e., small, cysteine-rich hydrophobic proteins (Figure 2). Hydrophobins are synthesized by filamentous fungi. The amino acid sequence of hydrophobins is highly evolutionarily conserved. Hydrophobins are classified into two classes based on the arrangement of cysteine residues, differences in solubility, and physical properties. Hydrophobins form amphipathic monolayers at hydrophobic-hydrophilic interfaces. Those proteins are involved in the formation of aerial hyphae, fruiting bodies, and spores as reviewed in [463,464]. It was shown that class I hydrophobins produced by *T. asperellum* enabled adhesion to cucumber roots. The authors hypothesized that hydrophobins protect hyphae against antimicrobial compounds were secreted by the host during colonization [244]. Inoculation of tomato and cucumber with a *T. harzianum* mutant with a deletion in the gene encoding hydrophobin showed that the mutant fungi were able to colonize the roots of both plants; however, the lateral roots were significantly shorter than those present in plants inoculated with the wild-type fungus [245]. Further penetration of roots by hyphae is possible due to fungal proteolytic enzymes, e.g., aspartyl protease (PapA) [465], and cellulolytic enzymes, e.g., endopolygalacturonase (ThPG1) [458], and arabinofuranosidases (Abf1, Abf2) [465] that allow for degradation of the plant cell wall. Another important element allowing *Trichoderma* for tissue penetration are swolenins (Figure 2), i.e., proteins possessing a cellulose-binding domain (CBD), similar to plant proteins—expansins. Swollenins disrupt the structure of the cellulose which results in changes to the plant cell wall architecture and the expansion of intercellular space [466]. Swollenins facilitate penetration of apoplast by the hyphae and give fungi an advantage during the competition for the niche with other microbes. Brotman et al. [250] reported that *T. asperellum* overexpressing swollenin showed a significantly improved ability to colonize cucumber roots, whereas swolenin knockout mutants showed a reduced ability to colonize roots. Moreover, it was found that the CBD domain acts as the MAMP, and can induce plant defense against *B. cinerae* and *P. syringae*. Similarly, *T. atroviride* overexpressing the swolenin-coding gene *Taswo1* improved the colonization rate and enhanced the growth of tomatoes and peppers. Moreover, the induction of the plant immune system was stronger by mutants overexpressing swollenins than by knockouts and wild-type fungi [467].

Sucrose plays important role in plant colonization by *Trichoderma*. As demonstrated by Macías-Rodríguez et al. [468], the concentration of carbohydrates, mainly arabinose, xylose, myo-inositol, fructose, and glucose in root exudates of *L. esculentum* is higher before colonization by *T. atrioviride*. Colonization of the root by *T. atrioviride* changed the exudation pattern and sucrose became a major component of exudates. Root-derived sucrose specifically enables better growth of fungi belonging to *Trichoderma,* since AMF fungi prefer glucose and fructose as a source of carbon. Analysis of the proteome of maize inoculated with *T. harzianum* T22 showed that 40 proteins involved in carbohydrate/starch metabolism were upregulated and 13 proteins were downregulated, which suggests that *T. harzianum* is able to modulate carbohydrate metabolism in colonized roots [413]. *T. virens* was shown to produce invertase that hydrolyses plant-derived sucrose. The sucrolytic activity of fungal cells is crucial for root colonization but also to increase the photosynthetic rate in maize leaves [409].

## Figures and Tables

**Table 1:** Examples of plant growth-promoting microorganisms. The effect of PGPM on plants and, if known, the mode of action of microorganisms is also included.

PGPM	Plant	Remarks	References
Bacteria			
***Acinetobacter* sp. RG30, *Pseudomonas putida* GN04**	*Zea mays*	Plant: - increased tolerance to Cu - enhanced chlorophyll content - increased Cu concentration in tissues Bacteria: - IAA synthesis - production of siderophores - solubilization of Cu and P	[17]
***Acinetobacter* sp. RSC9**	*Saccharum* sp.	Plant: - under salt stress enhanced number of leaves, fresh, dry weight, and germination ratio Bacteria: - IAA production - P, K, and Zn solubilization - N_2_ assimilation	[18]
***Agrobacterium* sp. 10C2**	*Phaseolus vulgaris*	- increased nodule formation - higher plant biomass - enhanced content of P, polyphenols, and flavonoids in grains - changes in the structure of the microbial community	[19]
***Arthrobacter globiformis***	*Zea mays,* *Triticum aestivum*	Plant: - enhanced biomass, uptake of Fe and P, and higher chlorophyll content under iron-stress Bacteria: - siderophores production	[20]
***Arthrobacter* sp.,** ***Bacillus megaterium***	*Lycopersicon esculentum*	- enhanced seed germination ratio, seedling length, and dry and fresh weight under salt stress	[21]
***Azospirillum brasilense***	*Cicer arietinum*	- increased resistance to *Ascochyta rabiei* via induction of plant defense-related genes (*Snakin2* and *DEF0422*)	[22]
***Azospirillum lipoferum***	*Triticum aestivum*	- improved germination, plant growth, higher chlorophyll content, and improved membrane stability under salt stress - increased production of SOD and osmolytes, i.e., proline, soluble protein, and sugars under salt stress	[23]
***Azotobacter* spp.**	*Zea mays*	Plant: - increased shoot dry weight, chlorophyll content, and N, P, Fe concentration under drought stress Bacteria: - production of siderophores	[24]
***Bacillus amyloliquefaciens***	*Zea mays*	- increased tolerance to salt stress, enhanced content of chlorophyll, soluble sugars, and glutathione, higher peroxidase/catalase activity	[25]
***Bradyrhizobium* sp., *Rhizobium leguminosarum, Azotobacter* sp.**	*Gossypium hirsutum*	Plant: - increased rate of seedling emergence, biomass, and N uptake Bacteria: - IAA production	[26]
***Burkholderia phytofirmans* PsJN**	*Triticum aestivum*	- improved water content and CO_2_ assimilation rate, water use efficiency, chlorophyll content, and higher yield under drought stress - improved ionic balance, antioxidant levels, higher N, P, K, and protein content in grains	[27]
***Burkholderia tropica***	*Lycopersicum esculentum*	Plant: - increased yield Bacteria: - N-fixation and P solubilization	[28]
***Enterobacter cloacae***	*Spinacia oleracea*	- protection against Fusarium wilt (*Fusarium oxysporum*)	[29]
***Frankia* spp.**	*Casuarina glauca, Casuarina equisetifolia*	- salt stress alleviation, higher dry biomass, chlorophyll, and proline content	[30]
***Methylobacterium* sp. 2A**	*Arabidopsis thaliana, Solanum tuberosum*	Plant: - the alleviation of salt stress of *A. thaliana*, with higher lateral roots density, number of leaves, and larger rosette diameter - reduced necrotic lesions and chlorosis in *S. tuberosum* infected with *P. infestans* Bacteria: - production of IAA, P solubilization, biocontrol activity against *Phytophtora infestans*, *Botrytis cinerea,* and *Fuasrium gramiearum*	[31]
***Pseudomonas putida***	*Lycopersicum esculentum*	Plant: - increased plant height, stem diameter, radical volume, dry biomass, and fruit yield Bacteria: - production of IAA	[32]
***Pseudomonas* sp. DW1**	*Solanum melongena*	- salt stress ameliorating effect, with increased dry weight, and seed germination - higher SOD activity in leaves	[33]
***Pseudomonas stutzeri* ISE12**	*Brassica napus*	- enhanced growth under salt stress, with a decrease in non-enzymatic antioxidants accumulation - improved seed germination ratio, number of leaves, chlorophyll content, and dry weight	[34]
***Rhizobium leguminosarum, Rhizobium* sp., *Bradyrhizobium* sp.**	*Oryza sativa*	Plant: - increased yield and uptake of N, P, K, and Fe - improved seed vigor, dry biomass, and leaf area with faster seedling emergence Bacteria: - production of IAA	[35,36]
***Serratia marcescens***	*Solanum melongena*	- salt stress alleviation, decreased Na+/Cl- content in leaves, lower lipid peroxidation level, and higher activity of antioxidant enzymes - enhanced biomass, longer stems, and bigger leaf area	[37]
***Serratia proteamaculans, Pseudomonas putida, Pseudomonas aeruginosa***	*Triticum aestivum*	Plant: - salt stress alleviation with enhanced plant height, root length, and yield, and higher chlorophyll content Bacteria: - ACC deaminase production	[23]
***Streptomyces* sp.**	*Arabidopsis thaliana, Lycopersicon esculentum*	Plant: - salt stress alleviation with increased biomass, chlorophyll content, and decreased proline content Bacteria: - production of IAA, ACC deaminase, P, and NaCl solubilization	[38]
***Streptomyces* sp.**	*Medicago sativa*	- protection against root-lesion nematode (*Pratylenchus penetrans*)	[39]
**Fungi**			
***Alternaria solani* IA300**	*Capsicum annum*	- enhanced number of leaves, flowers, dry, and fresh weight	[40]
***Apergillus niger* 9-p**	*Phasoleus vulgaris*	Plant: - increased biomass Fungus: - production of IAA, ACC deaminase, siderophores, protease, amylase, pectinase, xylanase, and P solubilization	[41]
***Aspergillus fumigatus***	*Glycine max*	Plant: - salt stress alleviation with enhanced biomass, leaf area, chlorophyll content, and photosynthetic rate - increased isoflavones, proline, SA, and JA content and lower ABA content Fungus: - GAs production (GA4, GA9, GA12)	[42]
***Collybia tuberosa, Clitocybe* sp., *Laccaria laccata, Hebeloma mesophaeum, Cyathus olla***	*Brassica napus*	Plant: - enhanced root and shoot growth, number of leaves, and biomass Fungi: - production of IAA	[43]
***Funneliformis mosseae, Ensifer meliloti***	*Vitis vinifera*	- enhanced plant height and dry weight - higher VOCs content in roots	[44]
***Fusarium equiseti, Glomus mosseae***	*Cucumis sativus*	- protection against anthracnose (*Colletotrichum orbiculare*) and damping off (*Rhizoctonia solani*) - enhanced shoot dry weight	[45]
***Fusarium verticillioides, Humicola* sp.**	*Glycine max*	- salt stress alleviation with increased shoot length, protein content, carotenoid, salicylic acid (SA), and enhanced SOD activity - decreased ABA level and lipid peroxidation level	[46]
***Glomus intraradices, Glomus mosseae***	*Olea europaea*	- enhanced yield, dry weight, height, stem diameter, and root length	[47]
***Lecanicillium psalliotae***	*Elettaria cardamomum*	Plant: - enhanced shoot and root length, biomass, and number of leaves - higher chlorophyll content Fungus: - production of IAA, ammonia, siderophores, and cell-wall degrading enzymes - P and Zn solubilization	[48]
***Mortierella antarctica, Mortierella. Verticillata***	*Triticum aestivum*	Plant: - enhanced fresh weight Fungi: - production of IAA, GA, and ACC deaminase	[49]
***Mucor* sp.**	*Arabidopsis arenosa*	- heavy metal (Zn, Cd, Fe, Pb) stress alleviation with enhanced biomass, root hair growth, improved water, and P content - upregulation of genes involved in nutrient acquisition (*HRS1, SPX1, MGD2*), and metal homeostasis (*MTPA2, ZIP7, IREG2*, *IRT2*)	[50]
***Penicillium bilaii***	*Pisum sativum*	- increased root dry weight, length, and P content in the shoot	[51]
***Penicillium* sp., *Penicillium radicum, Penicillium bilaiae***	*Medicago lupulina, Lens culinaris, Triticum aestivum*	- enhanced shoot growth and dry weight, and increased P uptake	[52]
***Phoma* sp.**	*Cucumis sativus, Arabidopsis thaliana*	- protection against cucumber mosaic virus (CMV) via ISR - higher number of leaves, increased fresh/dry weight, and the yield of cucumber	[53]
***Phoma* spp., *Trichoderma asperellum, Fusarium equiseti, Penicillium simplicissmum***	*Allium cepa*	- protection against white rot disease (*Sclerotium cepivorum*) with enhanced plant height, dry weight, and bulb perimeter - enhanced levels of peroxidase and polyphenol oxidase - upregulation of plant defense genes (*PR1*, *PR2*)	[54]
***Purpureocillium lilacinum, Purpureocillium. lavendulum, Metarhizium marquandii***	*Zea mays, Phaseolus vulgaris, Glycine max*	Plant: - enhanced plant height and biomass and N content in roots (*Z. mays*) and P in shoots (*P. vulgaris*) Fungi: - P solubilization and IAA production	[55]
***Trichoderma hamatum, Trichoderma harzianum, Trichoderma viride***	*Freesia refracta*	- accelerated flowering and enhanced development of lateral inflorescence shoots - increased K, Fe, Mn, and Zn uptake	[56]
***Trichoderma harzianum***	*Curcuma longa*	Plant: - enhanced plant height and yield Fungi: - biocontrol activity against rhizome rot and leaf blight (*Pythium aphanidermatum*, *Rhizoctonia solani*) - production of IAA, HCN, cellulase, and P solubilization	[57]
***Trichoderma phayaoense***	*Cucumis melo*	Plant: - enhanced plant development, biomass, and fruit yield Fungus: - biocontrol activity against gummy stem blight pathogens (*Stagonosporopsis cucurbitacearum*, *Fusarium equiseti*)	[58]
***Trichoderma viride***	*Brassica napus*	- enhanced biomass, lateral roots development, and germination ratio - changes in microbial composition	[59]
**Algae**			
***Anabaena oryzae, Anabaena doliolum, Phormidium fragile, Calothrix geitonos, Hapalosiphon intricatus, Aulosira ferilissima, Tolypothrix tenuis, Oscillatoria acuta, Plectonema boryanum***	*Oryza sativa*	- enhanced shoot and root length and biomass - improved protein, phenolics, flavonoids, and chlorophyll content - a higher activity of enzymes (peroxidase, phenylalanine, and ammonia lyase) - elevated levels of IAA, and IBA	[60]
***Anabaena variabilis, Anabaena laxa***	*Lycopersicon esculentum*	- protection against Fusarium wilt (*F. oxysporum*) with significant enhancement of growth, yield, and fruit quality - increased N, P, and Zn concentration - increased activity of defense enzymes (phenylalanine ammonia-lyase, polyphenol oxidase), increased activity of chitosanase, and β-1,3-glucanase	[61]
***Calothrix elenkinii***	*Oryza sativa*	- enhanced root/shoot length and fresh weight - improved chlorophyll and IAA content - higher nitrogenase and CMCase activity - 10-fold increase in microbiome population abundance	[62]
***Calothrix*** **sp., *A. laxa*, *Anabaena torulosa, Anabaena azollae, Anabaena oscillarioides***	*Triticum aestivum*	- enhanced biomass - nitrogen-fixing potential - higher endoglucanase activity	[63]
***Chlorella fusca***	*Cucumis sativus*	- protection against anthracnose (*Colletotrichum orbiculare*) via the induction of SAR	[64]
***Chlorella oocystoides, Chlorella minutissima***	*Zea mays*	- enhanced chlorophyll, P, and K content - improved biomass	[65]
***Chlorella vulgaris***	*Telfairia occidentalis*	- enhanced germination ratio - higher number of leaves and yield - improved chlorophyll, carbohydrates, proteins, and lipid content	[66]
***Microcystis aeruginsa***	*Oryza sativa*	- heavy metal (Cd) stress alleviation with decreased Cd accumulation, increased translocation of Cd from root to shoot, and enhanced dry weight	[67]
***Nostoc* sp.**	*Triticum aestivum, Oryza sativa*	Plant: - enhanced biomass and shoot/root length Algae: - production of IAA and zeatin	[68]
***Nostoc* sp.**	*Zea mays*	- enhanced dry mass - higher N content - production of exopolysaccharide	[69]
***Scenedesmus quadricauda, Chlorella vulgaris***	*Lycopersicon esculentum*	- enhanced biomass and root length	[70]
***Spirulina platensis***	*Zea mays*	- cadmium stress alleviation with improved photosynthetic electron flows and increased non-photochemical quenching - enhanced seed germination, shoot length, root fresh weight, and bigger leaf area - decreased Cd accumulation in shoot	[71]
**Mixed inoculants**			
***Anabaena* ssp., *Calothrix* sp., *Providencia* sp.**	*Triticum aestivum*	- enhanced yield and Fe, Cu, Zn, Mn, and protein content of grains	[72]
***Glomus fasciculatum, Bacillus subtilis***	*Tagetes erecta*	- enhanced flowering, with improved fresh weight and color of flowers	[73]
***Klebsiella variicola, Glomus multisubtensum, Rhizophagus intraradices***	*Helianthus tuberosus*	Plant: - enhanced biomass, yield, plant height, and leaf area - increased content of inulin in tubers Microbes: - P solubilization and IAA production	[74]
***Mesorhizobium mediterraneum, Rhizophagus irregulari***	*Cicer arietinum*	- enhanced yield and protein content of grain under water deficit conditions	[75]
***Rhizophagus intraradices, Glomus aggregatum, Glomus viscosum, Claroideoglomus etunicatum, Claroideoglomus claroideum, Pseudomonas fluorescens, Linum usitatissimum***	*Solanum lycopersicum*	- enhanced flower and fruit production, with increased lycopene, vitamins, sugars, and citric acid content of the fruits	[76]
***Rhizophagus intraradices, Pseudomonas* sp., *Bacillus* sp.**	*Sulla carnosa*	Plant: - enhanced biomass, stomatal conductance, photosynthetic pigment content, and photosynthesis rate under salt stress - increased proline content and higher activity of antioxidative enzymes Microbes: - production of IAA	[77]
***Septoglomus constrictum, Diversispora aunantia, Archaeospora trappei, Glomus versiforme, Paraglomus ocultum, Bacillus thuringiensis***	*Lavandula dentata*	Plant: - increased biomass under drought stress conditions, enhanced activity of the enzymatic antioxidant system, and enhanced nutrient uptake Microbes: - P solubilization, production of IAA, and ACC deaminase	[78]
***Trichoderma harzianum, Glomus* spp., *Pseudomonas fluorescens***	*Capsicum annuum*	- enhanced yield, higher activity of antioxidative, and defense enzymes	[79]

**Table 2:** Examples of mechanisms of plant growth promotion by PGPF.

Gene/Product	Function	Species	Reference
***aph*** **/acid phosphatase**	- increased P availability via phosphates solubilization	*Aspergillus, Trichoderma, Penicillium*	[238,239]
***AMT1; AMT2; AAT9/*** **ammonium transporter; amino acid transporter**	- improved N acquisition	*Tulasnella calospora*	[240]
***AQPF*** **/aquaporin**	- transport of water to the host - enhanced drought stress resistance	*Glomus intraradices*	[241]
***Phy*** **/phytase**	- increased P availability via solubilization of inositol	*Aspergillus, Trichoderma, Penicillium*	[238,239,242]
***acdS*** **/ACC deaminase**	- degradation of ethylene precursor and protects against elevated ethylene levels - ameliorates stress effects and promotes root growth	*Trichoderma asperellum, Penicillium citrinum, Trichoderma gamsii*	[243]
***Hyd; Qid*** **/hydrophobins**	- allows for adhesion of hyphae to the surface of roots and protects hyphae against antifungal compounds - functions as MAMP (microbe-associated molecular pattern) and triggers plant response involved with symbiont recognition	*Trichoderma asperellum, Trichoderma* *harzianum*	[244,245]
***MST2/*** **monosaccharide transporter2**	- development of arbuscules - facilitates root colonization	*Glomus* sp.	[246]
***Tex1*** **/*non-ribosomal peptided synthase***	- synthesis of trichovirin II (peptaibol) which activates the plant immune system	*Trichoderma virens*	[247]
***Thctf1*** **/*transriptional factor***	- regulates the synthesis of 6-pentyl-2H-pyran-2-one (6-PP) (VOC) which exhibits antifungal activity	*Trichoderma* sp.	[248]
***Thph1; Thph2/cellulases***	- cellulolytic activity - triggers plant immune system	*Trichoderma harzianum*	[236]
***sidD*/*siderophores synthase***	- synthesis of siderophores - improved Fe acquisition - defense against pathogens	*Trichoderma reesei, Trichoderma virens*	[249]
***Sm1; Sm2; Ep1; Swo*** ***/*** **cerato-platanins; swollenin**	- fungal elicitors and upregulation of genes involved in JA signaling (modulation of the immune system) - swollenin disrupts the plant cell wall structure and enables penetration of the apoplast	*Trichoderma citrinoviride, Trichoderma virens*	[250,251,252]

**Table 3:** Examples of mechanisms of plant growth promotion by PGPB.

Gene/Product	Function	Species	Reference
2,3-butanediol dehydrogenase	- synthesis of 2,3-butanediol - growth promotion - induction of ISR	*Bacillus* sp., *Aerobacter* sp., *Serratia* sp., *Enterobacter* sp., *Klebsiella* sp.	[253]
*acdS*/ACC deaminase	- degradation of ethylene precursor and protects against elevated ethylene levels - ameliorates stress effects	*Azospirillum* sp. *Pseudomonas putida*	[254,255]
alkaline phosphatase	- increased P availability via phosphates solubilization	*Pseudomonas brassicacearum*	[253]
*bud* operon	- synthesis of acetoine and 2,3-butanediol - induction of ISR (induced systemic resistance) - increased drought tolerance	*Enterobacter* sp638	[256]
chitinase; glucanase	- defense against fungal pathogens	*Pseudomonas aureginosa*, *Pseudomonas veronii*	[257,258]
exoprotease	- N acquisition - protection against pathogens	*Pseudomonas brassicacearum*	[253]
*fur*/transcription factor	- modulates gene expression encoding Fe transporter - Fe acquisition	*Pseudomonas brassicacearum*	[253]
*gcd*/pyrroloquinoline quinone (PQQ)-dependant dehydrogenase	- production of gluconic acid - P acquisition	*Pseudomonas fluorescens* F113, *Erwinia herbicola, Enterobacter intermedium*	[253]
*hcnABC*/HCN synthase	- protection against pathogens	*Pseudomonas fluorescens. Pseudomonas aeruginosa, Pseudomonas chlororaphis*	[259]
*ilvHI*; *ivlC*/acetohydroxyacid synthase; ketol-acid reductoisomerase	- synthesis of secondary metabolites including antibiotics - induction of ISR	*Bacillus subtilis*	[253]
*ipd; ppd* /indole-3-pyruvate decarboxylase; phenylpyruvate fenylopirogronian decarboxylase	- synthesis of IAA - promotion of root growth	*Azospirllum brasilense* Sp245, *Enterobacter cloacae* UW5, *Enterobacteriaceae*	[260,261,262]
nagA/N-acetylglucosamine-6 phosphate deacetylase	- chitinase-like protein and defense against fungal pathogens	*Pseudomonas brassicacearum*	[253]
*nif*/nitrogenase	- nitrogen assimilation	*Azospirllum, Burkoholderia, Rhizobium, Bradyrhizobium, Mesorhizobium,* *Delftia, Stenotrophomonas, Rhizobium, Brevundimonas,* *Variovorax, Achromobacter, Novosphingobium, Comamonas*	[263,264,265,266]
*phl* operon	- production of antibiotic 2,4-diacetylphloroglucinol - induction of ISR	*Pseudomonas fluorescens* F113, *Pseudomonas protegnes* CHA0	[267]
*phyC*/phytase	- increased P availability via solubilization of inositol	*Pseudomonas brassicacearum*	[253]
*rhb; rhtA*/siderophore synthase; mebrane Fe-regulated receptor	- production of rhziobactin (siderophore) and Fe acquisition - Fe uptake regulation	*Sinorhizobium meliloti*	[268]
*ribC*/riboflavin synthase	- growth promotion - defense against pathogens via ISR - upregulation of pathogenesis-related genes	*Pseudomonas yamanorum*	[269]
*yecA; speB*/polyamine permease; agmatinase	- synthesis and/or secretion of polyamines - lowers ethylene level in root cells - modulation of expansin genes expression - promotion of root growth - increases tolerance to low pH, oxidative and osmotic stress	*Bacillus subtilis* OKB105	[270]
